# Atlanta Society of Medicine

**Published:** 1886-09

**Authors:** 


### Society Reports.

### ATLANTA SOCIETY OF MEDICINE.

Officially Reported for THE ATLANTA MEDICAL AND SURGICAL JOURNAL.

STATED MEETING, July 20, 1886.

Vice-President N. O. Harris, M. D., in the chair.

Dr. W. P. Nicolson read a paper entitled

### "CONSTRICTION OF THE MEATUS AND SOME OF ITS RESULTING REFLEXES."

The doctor defined abnormal constriction as "any narrowing of the meatus, however slight, which caused nervous reflexes." He then gave a brief resumé of the anatomy of the normal urethra.

He took the position that, while many men go through life with a meatus much below the normal, without inconvenience, it was because the urethral mucous membrane was at no time subjected to continued irritation. On the other hand, a very slight constriction was frequently a source of much annoyance and discomfort, where a gonorrhœa or other irritant supervened. He had seldom been able to cure gonorrhœa, where there was much constriction, without enlarging the meatus. Even where a cure was thought to have been effected, there was a proneness to recurrence after excesses in alcholics or venery.

Again, it may first become manifest where the urine is too highly concentrated or is loaded with urates; or, where none of the above conditions exist, from decomposition of a drop or so of urine retained in the canal.

One of the most frequent and most annoying symptoms of this condition is irritation of the bladder with frequent micturition.

Another very common symptom is a burning sensation in the deep urethra. Seminal emissions are frequently due to constriction of the meatus and the resulting urethral irritation.

Several typical cases were reported, which had been promptly and permanently relieved by operations.

The doctor thought a number of the so-called kidney and bladder diseases owed their origin to this cause, and were curable by meatotomy.

In the doctor's experience, two, three and even four operations were occasionally necessary to a complete relief of the symptoms. He was convinced the chief reason why the operation was not more uniformly successful was because the incisions were not sufficiently free. The meatus should always be cut up to, or a little beyond, the normal caliber of the urethra, to prevent recontraction, which would otherwise very likely occur.

In the discussion which followed, Dr. J. McF. Gaston asked if divulsion or dilatation were never resorted to.

Dr. F. W. McRae asked Dr. Nicolson if he had ever seen urethral chills result from incision of the penile urethra.

Dr. W. F. Westmoreland, Jr., stated that several peculiar and interesting reflexes, other than those mentioned by Dr. Nicolson, had come under his observation. Two cases of epileptics, who had frequently three or four convulsions daily, had especially attracted his attention. Both patients were cured by operations. He thought the reflexes were due to spasm rather than constriction.

Dr. Henry Wile asked if Dr. Nicolson had ever seen a case of acne caused by constriction of the meatus; also, if enlarging the meatus did not render a man more liable to contract venereal diseases.

Dr. Virgil O. Hardon alluded to a boy, aet. nine years, whom he had seen several years ago. The boy had been choreic all his life, and was at that time unable to talk or walk. Examination revealed phimosis and constriction of the meatus. Operations afforded only partial relief. He stated that a similar condition was not uncommon in women; for the relief of which, "Emmet's button hole operation" is performed.

Dr. D. H. Howell asked Dr. Nicolson if he had ever cured a patient after cutting the meatus without introducing a bougie into the bladder.

In conclusion, Dr. Nicolson said experience had demonstrated that constrictions within two inches of the meatus were much more amenable to treatment by incision than either divulsion or dilatation; the latter often increased instead of relieved the irritability of the mucous membrane. He had never seen a chill from meatotomy where a bougie was not passed into the deep urethra. He never introduced a bougie into the bladder after this operation; only brought the instrument to a perpendicular; did not depress it between the legs.

